# Green barley mitigates cytotoxicity in human lymphocytes undergoing aggressive oxidative stress, *via* activation of both the Lyn/PI3K/Akt and MAPK/ERK pathways

**DOI:** 10.1038/s41598-019-42228-4

**Published:** 2019-04-12

**Authors:** Blanca E. Ruiz-Medina, Dennise Lerma, Michael Hwang, Jeremy A. Ross, Rachid Skouta, Renato J. Aguilera, Robert A. Kirken, Armando Varela-Ramirez, Elisa Robles-Escajeda

**Affiliations:** 1 The Cytometry, Screening and Imaging Core Facility & Border Biomedical Research Center & Department of Biological Sciences, The University of Texas at El Paso, El Paso, Texas USA; 2 Department of Biology, University of Massachusetts, Amherst, Massachusetts USA

## Abstract

Oxidative stress plays a critical role in numerous diseases. Therefore, the pursuit of compounds with antioxidant activity remains critical. Green barley young leaves aqueous extract (GB) was tested for its capacity to ameliorate cellular oxidative stress, and its potential cytoprotective mechanism was partially elucidated. Through Folin-Ciocalteau and 1,1-diphenyl-2-picrylhydrazyl (DPPH) colorimetric assays, GB total phenolic content and free radical scavenging activity were found to be 59.91 ± 2.17 mg/L and 110.75 µg/ml (IC_50_), respectively. Using a live cell-based propidium iodide dye exclusion assay and flow cytometry, GB was found to display significant cytoprotection activity on three human lymphocytic cell lines exposed to an aggressive H_2_O_2_-induced oxidative stress. The molecular mechanism for GB cytoprotection activity was assessed *via* bead-based xMAP technology on the Luminex platform and western blot analysis. GB treatment resulted in activation of Lyn, Akt, and ERK1/2, suggesting that GB is able to mitigate the H_2_O_2_-induced oxidative stress *via* activation of both the Lyn/PI3K/Akt and ERK/MAPK pathways. Our findings support the notion that GB extract has the potential to be a valuable therapeutic agent and may serve to establish a strategy to discover potential compound(s) or biological extracts/mixtures to be incorporated as a treatment to prevent oxidative stress-related diseases.

## Introduction

Oxidative stress plays a pivotal role in the initiation and progression of a plethora of diseases, including aging-associated illnesses, chronic inflammation, cardiovascular disease, diabetes, and oncogenesis. An adequate balance of free radicals is necessary for maintaining cellular homeostasis, and this is possible due to the intervention of antioxidants with free radical scavenger activity. The Plantae kingdom provides us with major amounts of antioxidants as part of our diet^1^. Therefore, focusing on the potential protective benefit of an extensive variety of chemical compounds with antioxidant effects, particularly those derived from fruits, vegetables, and edible plants is an ongoing important task^2^. Discovering plant-derived extracts with antioxidant activity remains a prominent endeavor in medicinal chemistry.

Young barley leaves extract, also referred to as green barley (GB), is widely consumed as a dietary supplement^3^. In addition to its vitamin and mineral content, studies have suggested that GB exhibits antioxidant properties and could help alleviate inflammatory diseases^4,5^. In the mid-1990s, a commercial GB (“Natural SOD”) available for human intake was fractionated, and three of those fractions were able to reduce the tumor necrosis factor-alpha (TNF-α) production/release in human monocytes THP-1 cell line^6^. A similar effect was observed in mononuclear cells isolated from peripheral blood and synovial fluid from patients afflicted with rheumatoid arthritis (RA)^4^. Around the same time, it was reported that a single compound isolated from green barley leaves exhibited antioxidant properties^5^. It was later clarified that the major flavonoid antioxidants in young green barley leaves were, in fact, the flavone-C-glycosides, saponarin, and lutonarin^7–9^. Further studies showed that the antioxidant activity obtained from a combination of saponarin/lutonarin (4.5:1 proportion) was comparable to that obtained from vitamin E (α-tocopherol) and butylated hydroxytoluene, two well known antioxidant compounds^10^. More recently, it was reported that gramine (also called donaxine), a natural indole alkaloid found in young barley *Hordeum vulgare* L. and other plant species, attenuates inflammation and cell proliferation in oral carcinogenesis involving the NF-κB and STAT3 pathway^11^. In addition, whole barley kernel extracts have been shown to exhibit antioxidant, antiradical, and antiproliferative capabilities on the colorectal cancer cell line Caco-2^12^. Other reports suggest that green biomass from young barley plants retains significant amounts of antioxidant enzymes like superoxide dismutase and catalase, as well as the non-enzymatic antioxidants vitamins C and E^13,14^. In agreement with these reports, a clinical study comprising 36 type 2 diabetic patients ingesting daily supplements of barley leaves in combination with antioxidant vitamins (C and E) conveniently decreased the low-density lipoprotein (LDL)-vitamin E content and blocked small dense-LDL oxidation, consequently decreasing some of the major risk factors of atherosclerosis and protecting type 2 diabetic patients against vascular diseases^15^. Lastly, GB has been shown to exert both antiproliferative and pro-apoptotic activities on human leukemia/lymphoma cells^3^.

In this study, we explored the potential prophylactic effect of GB on cells undergoing aggressive H_2_O_2_-induced oxidative stress. We also investigated the cell signaling implicated in the GB-cytoprotection activity. Overall, our data indicate that GB possesses potent free radical scavenger properties and attenuates H_2_O_2_-induced cell death. Our findings support the notion that the GB extract has the potential to be a valuable therapeutic agent in precluding oxidative stress-induced conditions.

## Materials and Methods

### Green barley extracts (GB) preparation

Aqueous green barley extracts (GB) were prepared as previously described^3,16^ by using dry powder from young leaves of *Hordeum vulgare* L., an herbaceous supplement sold as a proactive source of antioxidants, vitamins, and minerals (Vitamin World; www.vitaminworld.com). Briefly, rehydrated green barley powder suspensions in phosphate buffer saline solution (PBS) or double distilled water (ddH_2_0) were frozen (−80 °C) and thawed (room temperature) three times. The samples were then sonicated, centrifuged and filtered (0.2 µm pore size) for sterilization purposes. Typical volumes in microliters and their corresponding lyophilized dry weight values in mg/ml were measured as previously described^3^: 10 µl, 25 µl and 50 µl of GB are equivalent to 0.3 ± 0.009, 0.75 ± 0.006 and 1.5 ± 0.048 mg/ml of lyophilized powder, respectively.

### Quantification of GB total phenolic content using Folin-Ciocalteu reagent

**Figure 1:**
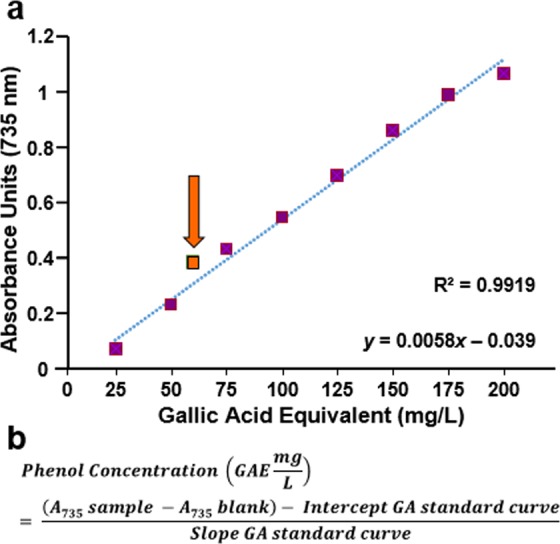
GB total polyphenol content of GB is 59.91 ± 2.17 mg/L gallic acid equivalents (GAE). (**a**) Correlation between total polyphenolic content and the absorbance (A_735nm_). The orange square corresponds to GB, 59.91 ± 2.17 mg/L GAE value, whereas purple squares to gallic acid working standards used as reference reagent. Linearity of calibration curve with correlation coefficient (R-squared, R^2^ = 0.9919) and linear equation are included in the graph (y = 0.0058 × −0.039). (**b**) The equation used to calculate the GAE value. Each data point represents the mean of four replicas. For each data point, the standard deviations of absorbance (A_735_ nm) were consistent with <0.072 (data not shown).

The amount of GB total phenolic content was determined by using the Folin-Ciocalteau colorimetric assay, which utilizes gallic acid as a standard reagent (modified from Ainsworth *et al*.)^17^. GB samples were prepared by mixing 17.1 µl of GB aqueous solution (30.85 ± 0.009 mg/ml of GB dry weight) with 82.9 µl of water, added with 0.2 ml of 10% v/v of Folin-Ciocalteau reagent (Sigma-Aldrich, St. Louis, MO), sonicated for 10 seconds and allowed to stand for 30 min in the dark at room temperature. Subsequently, 0.8 ml of 700 mM sodium bicarbonate aqueous solution was added to the reaction, sonicated for an additional 10 seconds and incubated 2 h as described above. An aliquot of 250 µl of each reaction mixture was transferred to a 96-well plate and the absorbance of each sample at 735 nm was determined by using a microplate-reader spectrophotometer (Tecan; Morrisville NC). The GB final concentration of 478.20 µg/ml of dry weight was reached to match the highest concentration used in the DPPH assay. For the reference reagent, a stock solution of 5 g/L gallic acid in methanol was utilized to prepare eight working standards, containing equivalent phenol concentrations of 25, 50, 75, 100, 125, 150, 175, and 200 mg/L gallic acid; an effective range to generate the calibration curves. An aliquot of 0.1 ml of each working standard was mixed with 0.2 ml of Folin-Ciocalteau reagent and then added with 0.8 ml of 700 mM sodium bicarbonate; sonication and incubation conditions were performed as above. A sample containing just water and reagents was used as a reference blank. The total phenolic content was expressed as milligrams of gallic acid equivalent (mg/L GAE). Both experimental and standard samples were performed in quadruplicates for each experiment. The gallic acid standard curve was created by plotting the average absorbance (A_735_) values for each data point (*y*-axis) versus mg/L of gallic acid equivalent (GAE; *x*-axis). The best-fit regression line was added to calculate the intercept and slope for the gallic acid standards. The equation utilized to obtain the total phenolic content in GB is depicted in Fig. 1b.

### GB antioxidant analysis using cell-free DPPH radical assay

To evaluate free radical scavenging activity of GB, the 2,2-diphenyl-1-picrylhydrazyl (DPPH; Sigma, St Louis, MO) free radical colorimetric assay was utilized by recording its absorbance spectrophotometrically^18^. To find the GB optimal concentration for this assay, an aqueous solution containing 30.85 ± 0.009 mg/ml of GB dry weight was tested at 1:2 serial dilutions mode (in methanol; Sigma); finding an optimal range from 485.8 µg/ml to 15.1 µg/ml. Solutions of DPPH free radical were prepared in methanol at a concentration of 1 mM (20X), followed by further dilution, also in methanol, to 50 µM (1X working solution). As antioxidants controls, solutions of reduced glutathione (L-Glutathione(GSH); Sigma) and vitamin E (α-tocopherol) and their 1:2 serial dilutions, prepared in methanol, were included in a range from 50 µM to 1.56 µM final concentration per reaction. Moreover, water and methanol samples were used as solvent control and blank, respectively. Aliquots of 200 µl per well containing GB extract in a 96-well solid-bottom plate, were added with 100 µl of 50 µM DPPH (1X) and incubated at room temperature for 1, 10, 20 and 30 minutes in the dark; DPPH final concentration per reaction (300 µl total volume) was of 16.66 µM. GSH and vitamin E were concomitantly processed in a similar manner. The DPPH absorbance at 517 nm was measured by using a microplate absorbance reader for 96-well plates (Tecan Systems, San Jose, California).

The percentage of antioxidant activity (AA%), equivalent to the percentage of DPPH purple color reduction, was determined by comparison with the absorbance of methanol-containing controls (blanks) and converted into the percentage of antioxidant activity by using the following equation:

$${\rm{AA}} \% =100-[\frac{AU\,{\rm{methanol}}-AU\,{\rm{of}}\,{\rm{extract}}}{AU\,{\rm{methanol}}}]\times 100$$

*AU* corresponds to *A*bsorbance *U*nits recorded at 517 nm. The rate (%) of DPPH∙ discoloration specifies the free radical scavenging efficiency of the experimental extract or chemical compound^19^. Values are reported as the average ± standard deviation (SD) of five independent experiments. The inhibitory concentration 50% (IC_50_) value for GB as a free radical scavenger was calculated by linear interpolation of the best-fit regression line. Here, IC_50_ is defined as the antioxidant concentration required to reduce 50% of the DPPH present in the reaction after 30 min of incubation.

### Cell lines and culture conditions

Three human lymphocytic cell lines were utilized; YT natural killer (NK)-like lymphoma^20–22^, pre-B acute lymphoblastic leukemia Nalm-6^23^ and mature T acute lymphoblastic leukemia Jurkat^24^; all cell lines were derived from a male donor^25^ (Table 1). The culture medium was routinely RPMI (HyClone, Logan UT, USA) supplemented with 10% heat-inactivated fetal bovine serum (HyClone) and 100 U/ml penicillin and 100 μg/ml streptomycin (Lonza, Walkersville, MD); also referred as complete medium. Cells were routinely incubated at 37 °C in humidified 5% carbon dioxide (CO_2_) atmosphere using a regular water-jacketed incubator. To secure high viability, before preparing an experimental multi-well plate, cells were processed as detailed previously^26^.

**Table 1:** Leukemia/lymphoma cell lines utilized in this study.

Cell Line^‡^	Tumor Type	Age of Donor*
Jurkat	Non-Hodgkin T-lymphoma	14
Nalm-6	Pre-B leukemia	19
YT	NK-like lymphoma	15

^‡^In-depth information for each cell line is available online from ATCC webpage (http://www.atcc.org/).

*Years old. All cell lines were derived from a male donor.

### H_2_O_2_-induced oxidative stress assay

Exponentially growing cells at 60–75% confluence were collected and seeded on 24-well plates at a density of 100,000 cells/well in 1 ml of complete media. After overnight incubation, cells were treated with 10 or 50 µl of GB and incubated another 18 h. Next, cells were collected and centrifuged (262 × g for 5 min) to remove supernatant, washed with pre-warmed (37 °C) complete media, resuspended with 1 ml of complete fresh media and plated in a 24-well plate. To induce oxidative stress, cells were exposed to 600 µM of H_2_O_2_ and incubated for an additional 24 h. Subsequently, cells were collected into an ice water pre-cooled flow cytometric tube, centrifuged and resuspended with a staining solution consisting of 400 μl of PBS containing 5 μg/ml of propidium iodide (PI), and incubated 1 min on ice protected from light. Control samples exposed to 600 µM H_2_O_2_ without GB pre-treatment were considered as the maximum toxicity possible and utilized as a reference to estimate the percentage of GB-protection effect. In addition, untreated cells and cells treated with 10 μl and 50 μl of PBS alone were included as controls. Both experimental samples, as well as controls, were handled identically and processed in parallel cultures. The cytotoxicity values are presented as mean ± standard deviation without any further normalization.

### Cytotoxicity analysis using live-cell and propidium iodide exclusion assay

Vital dye propidium iodide (PI) exclusion assay was used to monitor cytotoxicity *via* flow cytometer. Cells stained with the membrane-impermeant dye PI (5 µg/ml), a nucleic acid intercalator, were examined in a live-cell manner by using flow cytometric protocol. Viable cells with intact plasma membranes exclude PI. The main objective was to identify the distribution of PI-positive cells, with loss of plasma membrane integrity, which was considered dead cell subpopulation. Cells were collected in a flow cytometric tube, stained with PI, gently vortexed and immediately analyzed using a flow cytometer (Cytomics FC500; Beckman Coulter, Miami, FL). For each sample, a maximum of 10,000 events (cells) was acquired and data analyzed using CXP software (Beckman Coulter, Miami, FL). The cytotoxicity percentages obtained are expressed as a mean ± standard deviation.

### Luminex multiplex kinases analyses

Pre-B Nalm-6 cells growing as described above were seeded in a multi-well plate and treated with 25 and 50 µl/ml of GB for 3 h^3^. Subsequently, cells were washed with PBS and lysed with Milliplex MAP cell signaling lysis buffer containing a protease inhibitors cocktail (Millipore, Billerica, MA). The Milliplex MAP Human Src Family Kinase kit (Millipore, Billerica, MA) was used to detect tyrosine phosphorylation of Lyn (p-Tyrosine397), Yes (p-Tyrosine421) and Hck (p-Tyrosine411), by utilizing 25 µg of total protein per cell lysate following the manufacturer’s instructions (Millipore). Protein concentrations were determined using bicinchoninic acid (BCA) reagent (Thermo Scientific, Rockford, IL).

### Analysis of Akt, MAPK/ERK and STAT5 phosphorylation via Western blot

The phosphorylation of Akt was assessed *via* Western blotting as detailed previously^27^. Nalm-6 cells seeded in a 6-well plate format were exposed to 50 µl/ml of GB for 3 h, pelleted, and lysed. The total protein content per cell lysate was quantified by the BCA method (Thermo Scientific). Equal concentration of protein per lane was separated by SDS-PAGE and transferred onto a polyvinylidene fluoride (PVDF) membrane (Millipore). Western blot assays were developed with horseradish peroxidase-conjugated goat anti-rabbit IgG (heavy plus light chains; KPL) and visualized using enhanced chemiluminescence and X-ray film or the C-DiGit Blot Scanner (LI-COR). When reblotting, polyvinylidene difluoride membranes were incubated with stripping buffer (100 mM β-mercaptoethanol, 2% SDS, 62.5 mM Tris-HCl, pH 6.7) at 55 °C for 30 min, blocked, and then reprobed. The anti-phospho-p44/42 MAPK (ERK1/2) Threonine (Thr) 202/Tyrosine (Tyr) 204 polyclonal antibody, anti-p44/42 MAPK (ERK1/2) polyclonal antibody, anti-phospho-AKT Threonine (Thr) 308 monoclonal antibody, anti-phospho-AKT Serine (Ser) 473 monoclonal antibody, anti-AKT monoclonal antibody, and anti-phospho-STAT5 Tyrosine (Tyr) 694 antibody were purchased from Cell Signaling Technology (Danvers, MA). The anti-STAT5 antibody was purchased from BD Biosciences (San Jose, CA). All antibodies were used according to the manufacturer’s protocol. Densitometry analysis of Western blots was performed using the ImageStudio software suite (LI-COR).

### Statistical analysis

Each measurement was performed at least in triplicate. Data are displayed as average with its derived standard deviation to denote experimental variability. Two-tailed paired Student’s *t*-tests were performed to determine the statistical significance between the two sample groups. A value of P < 0.05 was deemed significant to indicate whether comparisons of two treatments possessed statistical significance.

## Results

### Phenolic content in GB

The analysis of soluble phenolic compounds in GB was performed by using an aqueous solution prepared from green barley dry powder dissolved in ddH_2_O. Polyphenols in plant extracts or other samples, react specifically with redox Folin-Ciocalteu reagent forming a blue complex that can be measured by spectrophotometry. The polyphenolic contents in GB are expressed in gallic acid equivalents (GAE). The linearity of the calibration curve exhibited a correlation coefficient (R-squared, R^2^) of R^2^ = 0.9919 (Fig. 1a). The total polyphenolic content in GB was of 59.91 ± 2.17 mg/L GAE (average ± SD), assessed by using the equation depicted in Fig. 1b.

### GB displays potent free radical scavenging activity

**Figure 2:**
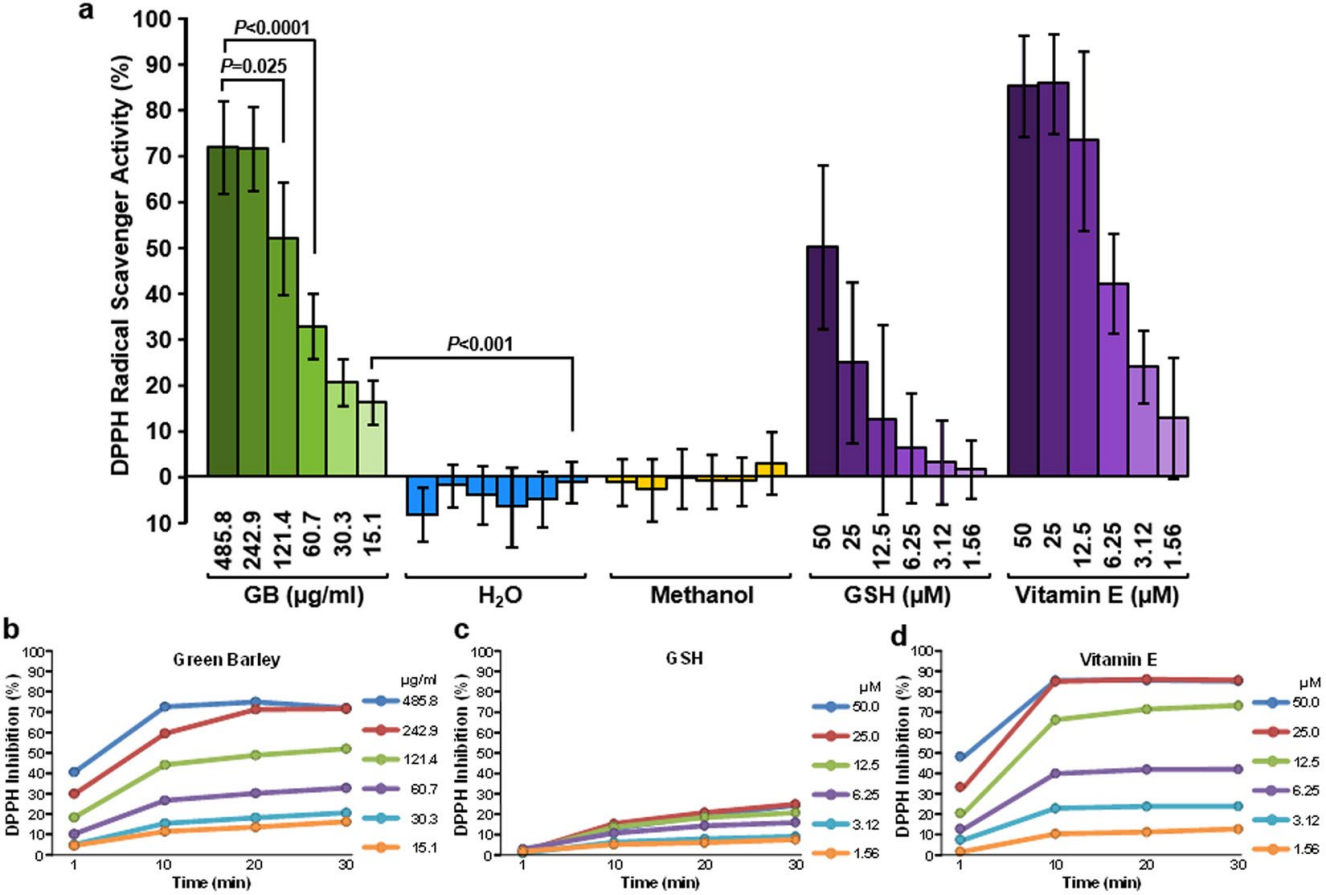
GB exhibits potent anti-oxidant activity evidenced by DPPH free radical scavenging assay in a dose and time-dependent manner. (**a**) A gradient of GB concentrations (485.8 µg/ml to 15.1 µg/ml) were incubated with DPPH for 30 min at room temperature, and the absorbance at 517 nm was recorded. DPPH radical scavenging activity was calculated according to the equation described in materials and methods. Glutathione (GSH) and vitamin E (50 to 1.56 µM) were included as positive antioxidant controls and analyzed concomitantly. Water was used as a solvent control, whereas methanol was used as a blank. Data are presented as the mean ± standard deviation of five independent experiments. Two-tailed paired Student’s *t*-tests were accomplished to determine the statistical significance of two experimental samples (*P* values). GB serial dilution concentrations (1:2; 485.8 to 15.1 µg/ml) were incubated with DPPH for 1, 10, 20 and 30 min at room temperature and the absorbance at 517 nm was recorded. Glutathione (GSH) (**c**) and vitamin E (50 to 1.56 µM) (**d**) were included as positive antioxidant controls and concurrently analyzed. Data are shown as the  average of five independent experiments.

The conversion of DPPH∙ (2,2-diphenyl-1-picrylhydrazyl) free radical to DPPH (diphenyl-picryl hydrazine) reaction was utilized to evaluate the GB biological antioxidant capacity *in vitro*. GB exhibited a scavenging effect of 71.83% and 16.15% at the maximum (485.8 µg/ml) and minimum (15.1 µg/ml) concentration tested, respectively (Fig. 2a). The well-known antioxidants, glutathione (GSH) and vitamin E were tested from 50 to 1.56 µM concentrations, and as expected, both GSH and vitamin E revealed scavenging effect, being vitamin E most effective than GSH (Fig. 2b,c). The GB antioxidant pattern was comparable to vitamin E (Fig. 2a). In contrast, water and methanol failed to display antioxidant activity (Fig. 2a). Additionally, to explore whether the GB scavenging activity was a time-dependent occurrence, a GB serial dilution concentrations (1:2) were incubated with DPPH free radical as above for 1, 10, 20 and 30 min at room temperature and the absorbance was quantified (Fig. 2b). GSH and vitamin E were also analyzed. At 30 min of incubation, vitamin E was found to be ~3.5 fold times more efficient at 50, 25 and 12.5 µM concentration than GSH (Fig. 2c,d). The GB scavenging activity resembled that seen with vitamin E, instead of GSH (Fig. 2b–d). GB and vitamin E reached their substantial free radical scavenger activity after the first 10 min of incubation with minimum changes observed after (Fig. 2b,d). The IC_50_ value for GB scavenging activity was of 110.75 µg/ml (Fig. 2b), whereas vitamin E was of 7.552 µM (Fig. 2d). The IC_50_ value for GSH could not be determined since the DPPH scavenge was of 24.73% at the maximum concentrations tested (Fig. 2c). Thus, GB was capable of effectively scavenging the DPPH free radical reagent, in a concentration- and time-dependent manner, suggesting that GB is a potent free radical scavenger.

### GB exhibits protective activity in human lymphoid cells upon oxidative stress

**Figure 3:**
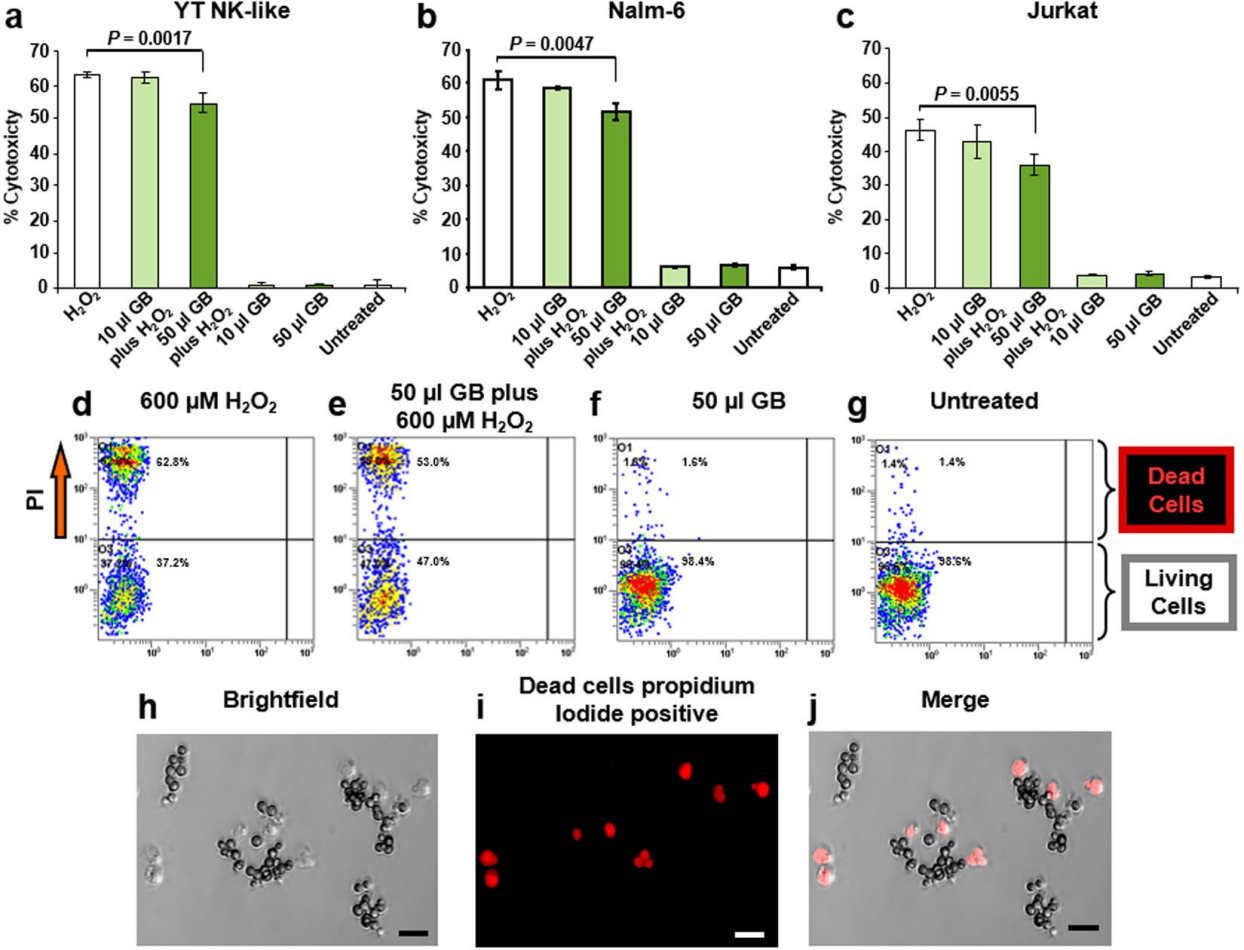
GB ameliorated H_2_O_2_-induced oxidative stress in lymphocytic cells. Graphs exhibiting the protection effect of GB against H_2_O_2_ (600 µM) aggression are in panels (a–c) which correspond to YT, Nalm-6 and Jurkat, respectively; each bar represents an average of four independent experiments and the error bar its standard deviation. Cells seeded in a 24 well plate were incubated with GB for 18 h, washed, exposed to 600 µM of H_2_O_2_, incubated for an additional 24 h period, stained with PI and processed via flow cytometry. Untreated cells, as well as cells exposed to 10 and 50 µl of GB alone or exposed to just 600 µM of H_2_O_2_, were included as controls. Cell viability was determined by using PI exclusion dye and flow cytometric assay. Data acquisition and analysis of PI-positive cells (dead) distributions were performed via CXP software (Beckman Coulter). Statistical significance between two samples was calculated using Student’s *t*-test (P value). Panels d-g show representative two parameter flow cytometric dot plots, where FL1 and FL2 detectors were plotted at x-axis versus y-axis, respectively. PI-stained YT cells (dead) are depicted in top left quadrat, whereas PI negative unstained cells (living) in the bottom left quadrant; H_2_O_2_-treated cells (**d**); GB-pretreated (50 µl) cells followed by exposure to H_2_O_2_ (**e**); cells treated with 50 µl of GB alone (**f**); and untreated cells (**g**). The various dot (event/cells) color in each plot, designates a density gradient; low-density, blue dots, whereas high-density, red dots. An alternative strategy to quantify the cytotoxicity *via* vital dye PI exclusion assay using fluorescent microscopy in YT cells is illustrated in panels (h–j); bright field DIC image (**h**); cells exhibiting red fluorescence signal with compromised plasma membranes, PI positive (**i**); and a merged image of bright field and red channel (**j**). Scale bar = 20 µm.

A panel of leukemia/lymphoma cell lines was selected to review the cytoprotection effect of GB. The cytotoxicity percentages were measured by using vital dye PI exclusion assay monitored by flow cytometric protocol (Fig. 3). This approach identifies *in vivo* at a single-cell level PI-positive cells, embracing the cell population with plasma membrane damage, which is considered as dead cells^28^. Cells exposed to 10 µl/ml of GB (equivalent to 0.3 ± 0.009 mg/ml of GB dry weight) displayed moderate cytoprotection after exposure to H_2_O_2_ (Fig. 3a–c), however, based on the P value, a trend in this protective effect is observed. The three lymphoid cell lines pre-incubated with 50 µl/ml of GB (equivalent to 1.5 ± 0.048 mg/ml of GB dry weight) consistently exhibited a significant reduction in mortality after treatment with H_2_O_2_ (600 µM). YT, Nalm-6, and Jurkat cells showed a 13.29%, 15.5%, and 22.07% reduction in mortality respectively compared to the H_2_O_2_ control, with *P* values oscillating from 0.0017 to 0.0055 (Fig. 3a–c). No difference was observed on cells left untreated or treated with 10 µl/ml or 50 µl/ml of GB, indicating that the extract alone is not an influential factor in cell viability. The H_2_O_2_ insult (600 µM) resulted in less toxicity on Jurkat cells (46.2 ± 2.98%) whereas YT cells (62.97 ± 0.72%) were to some extent the most sensitive to this oxidative stress aggression (Fig. 3a–c). Representative flow cytometry dot plots are shown in Fig. 3d–g. An alternative strategy to quantify the cytotoxicity *via* vital dye PI exclusion assay using fluorescent microscopy is illustrated in Fig. 3h–j. Thus, GB exerted a consistently protective effect on the three human lymphoid cells undergoing oxidative stress on living cells.

### GB enhances Lyn tyrosine kinase phosphorylation

**Figure 4:**
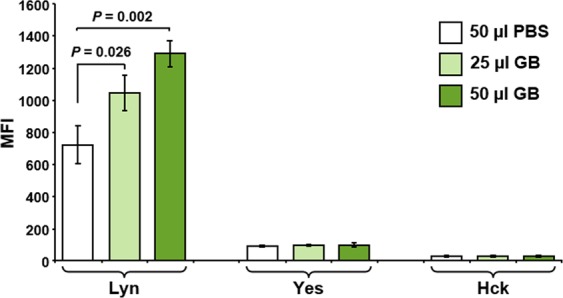
GB induced phosphorylation of Lyn tyrosine kinase in pre-B Nalm-6 cells. Protein extracts from pre-B Nalm-6 cells, treated with 25 and 50 µl/ml of GB for 3 h, were analyzed for activation of Src family kinases, Lyn, Yes and Hck, using the bead-based Luminex xMAP platform. Cells treated with 50 µl of PBS solvent control were concurrently analyzed. The median fluorescence intensity (MFI) is plotted in the y-axis; whereas the three Src family kinases are plotted in the x-axis. Each bar represents the average of three experiments and the error bars are their associated standard deviations. Statistical variability was determined using Student’s *t*-test. The 25 and 50 µl of GB correspond to 0.75 ± 0.024 and 1.5 ± 0.048 mg/ml of GB lyophilized powder, respectively. Data acquisition, processing, and analysis were achieved using xPONENT 3.1 software (Luminex).

A panel of Src tyrosine kinases involved in cell signaling was analyzed to elucidate whether those enzymes are implicated in the GB cytoprotection activity. Nalm-6 cells were exposed to GB and protein extracts were utilized to assess the activation of Lyn, Hck, and Yes. For this purpose, a bead-based multiplex technology and antibodies recognizing specific tyrosine phosphorylation sites, Lyn (Tyr397), Hck (Tyr411) and Yes (Tyr421) were utilized. Out of the three kinases, only Lyn was found to be activated by GB. Treatment of cells with 25 µl/ml and 50 µl/ml of GB resulted in a 40% (*P* = 0.026) and 70% (*P* = 0.002) increase in Lyn phosphorylation respectively compared to PBS-treated control (Fig. 4). In contrast, changes in phosphorylation levels of Hck (Tyr411) and Yes (Tyr421) kinases were not detected when compared with both PBS- and GB-treated cells (Fig. 4). Thus, GB stimulates the phosphorylation of Lyn (Tyr397) in pre-B Nalm-6 cells in a concentration-depended modality, suggesting its potential participation as an antioxidative stress kinase.

### GB induces activation of Akt and ERK1/2

**Figure 5:**
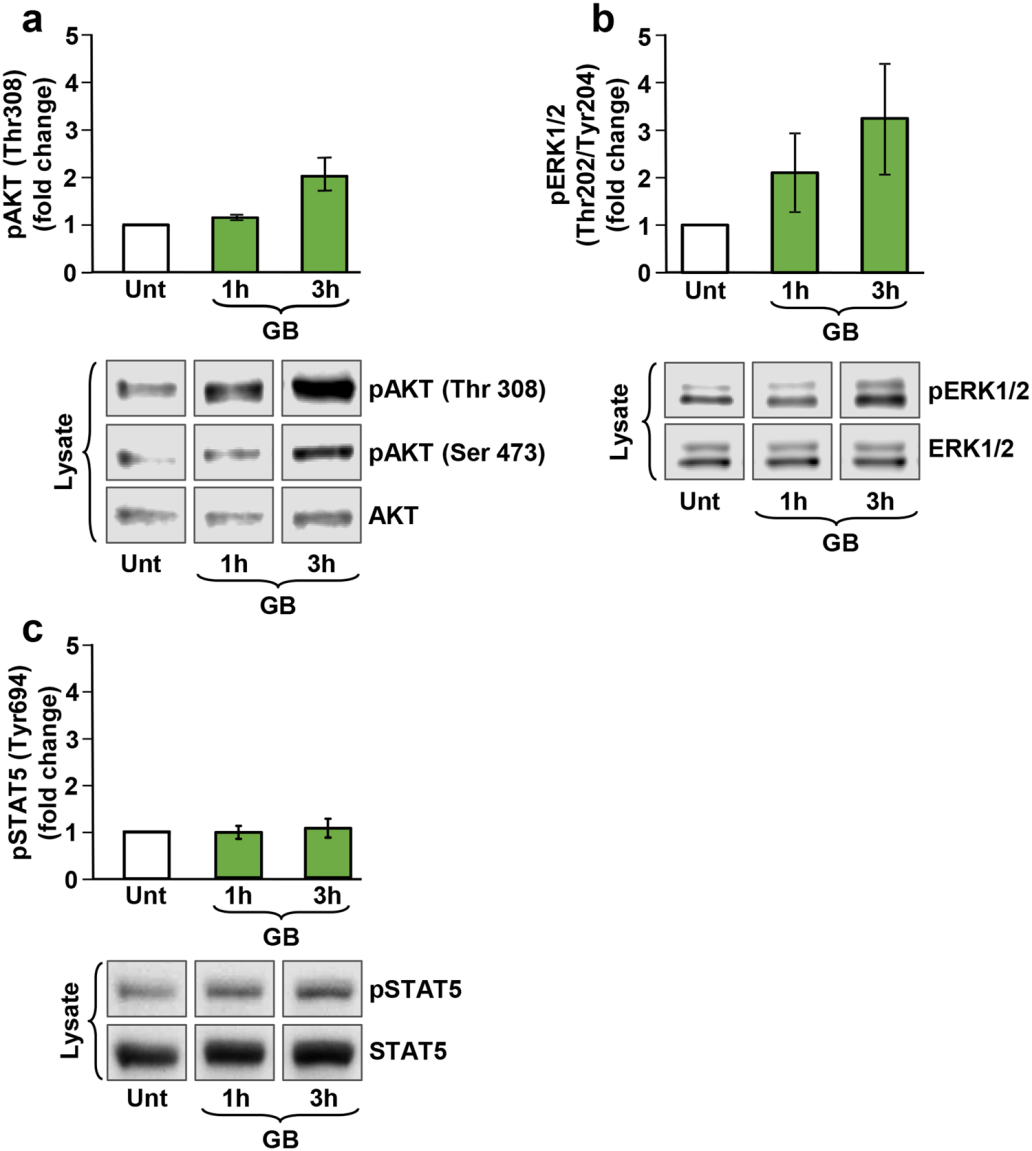
GB elicited phosphorylation of both Akt and MAPK/ERK kinases in pre-B Nalm-6 cells in a time-dependent manner. Whole cell lysates were subjected to Western blot analysis with the antibodies indicated in material and methods. (**a**,**b**) The bar graphs represent densitometry analyses showing a comparable increase in pAkt (Ser 473) and pMAPK/ERK phosphorylation induced after 1 h and 3 h of GB-treated cells (50 µl), respectively (n = 2). As a reference control, lysates from untreated (Unt) pre-B Nalm-6 cells were included, and also, to establish the basal levels of Akt and MAPK/ERK phosphorylation; representative Western blots displaying GB-stimulated pAkt (Ser 473), pAkt (Thr 308) and pERK1/2 phosphorylation are shown in the lower panels. (**c**) The bar graph represents densitometry analysis showing a comparable increase in STAT5 (Tyr 694) phosphorylation caused after 1 h and 3 h of GB-treated cells (50 µl), respectively (n = 3). As a reference control, lysates from untreated (Unt) pre-B Nalm-6 cells were included; western blots are displaying GB-stimulated STAT5 phosphorylation, as compared with total STAT5 protein are shown in (**c**), lower panel. The 50 µl of GB correspond to 1.5 ± 0.048 mg/ml of GB lyophilized powder.

The activation of Akt and p44/42 mitogen-activated kinase (MAPK), also known as extracellular signal-regulated kinase (ERK1/2) enzymes are necessary for transduction of the Lyn/PI3K/Akt and MAPK/ERK signaling pathways. Phosphorylation of these enzymes serves as robust markers involved in pro-survival cell signaling^29,30^. To investigate the potential involvement of Lyn/PI3K/Akt and MAPK/ERK pathways on GB protective effect, the phosphorylation levels of Akt and ERK1/2 were determined using phosphospecific antibodies to identify the active forms of both enzymes. Pre-B Nalm-6 cells were left untreated or exposed to GB for 1 and 3 h, and whole cell lysates were examined concomitantly *via* Western blot. As seen in Fig. 5, GB treatment resulted in a modest increment of Akt (Thr308) phosphorylation after 1 h. However, phosphorylation reached ~2 fold increase after a 3 h exposure (Fig. 5a). A similar phosphorylation pattern was detected for pAKT (Ser473), which is also indicative of AKT activation (Fig. 5a, lower panel). In addition, ERK1/2 phosphorylation increased ~2 fold after 1 h of GB treatment and reached ~3 fold increase at 3 h compared to the non-treated cells (Fig. 5b). Therefore, GB treatment resulted in activation of the Lyn/PI3K and MAPK/ERK pathways in a time-dependent manner which could contribute to diminishing cell death after oxidative stress.

Another protein that plays a prominent role in proliferation and cell survival is the signal transducer and activator of transcription (STAT) 5^31^. It’s been previously reported that the Janus kinase 2 (JAK2)/STAT5 pathway plays a crucial role in the survival and proliferation of chronic myeloid leukemia cells obtained from patients^32^. Thus, pre-B Nalm-6 cells were treated as described above and protein extracts investigated for activation of STAT5 *via* Western blot analysis. As seen in Fig. 5c, there was a slight increase (1.3 fold) in STAT5 phosphorylation compared to the non-treated control. Therefore, GB was able to modestly induce STAT5 phosphorylation, suggesting that the Lyn/PI3K and MAPK/ERK pathways play larger roles on GB effects than JAK2/STAT5.

## Discussion

Polyphenols are potent and the most abundant antioxidants in the diet, mainly supplied by fruits, vegetables and plant-derived beverages such as juices, tea, coffee, and red wine^33^. These compounds are effective in preventing inflammatory and cardiovascular diseases by neutralizing the damage caused by free radicals. The objective of this study was to explore whether an aqueous green barley extract (GB) contains polyphenols with the capacity to scavenge free radicals and exert its antioxidant protective activity on a panel of human lymphoid cells undergoing to H_2_O_2_-induced oxidative stress. The GB extract was found to have a total phenolic content of 59.91 ± 2.17 mg/L GAE, and its DPPH radical-scavenging activity was found to be 110.75 µg/ml (IC_50_). In addition, GB treatment resulted in activation of the pro-survival pathways Lyn/AKT and MAPK/ERK, and the reduction of H_2_O_2_-induced mortality. These findings suggest that GB could be an attractive source of antioxidants.

The antioxidant and anticancer activities in fruits and vegetables are attributable to the synergistic effect of their complex mixture of phytochemical components^34^. For instance, whole extracts of the “Red Delicious” apples (*Malus pumila*) had higher antioxidant activity and inhibited the proliferation of cancer cells more efficiently than apple extracts without skin^35^. Moreover, total polyphenol extracts from cranberries (*Vaccinium macrocarpon* Ait. Ericaceae) displayed more elevated anti-inflammatory and antiproliferative activity as compared with its individual components^36^. Studies have shown that extracts from mango (*Mangifera indica* L.), quince (*Cydonia oblonga* Miller) and banana (*Musa paradisica*) exerted protection on H_2_O_2_-induced oxidative toxicity in erythrocytes^37–39^. In addition, papaya (*Carica papaya* L.) epicarp extracts and date (*Phoenix dactylifera*) seed oil ameliorate the H_2_O_2_-induced oxidative stress insult *in vitro* in neuronal SH-SY5Y cells or normal human epidermal melanocytes, hypothesizing potential utility in patients with neurological disease or as a chemopreventive agent in melanocyte-related pathologies, respectively^40,41^. Plant antioxidants may decrease carcinogenesis by neutralization of ROS or other free radicals that can cause structural DNA damage, therefore decreasing the risk of tumor formation^42^. However, in the light of recent evidence, antioxidants should be considered cautiously in cancer afflicted patients, due that once that the cancer is established, an antioxidant therapy could favor cancer exacerbation^43^ and metastasis development^44^.

The total phenolic content of extracts or plants contributes to their antioxidant activity. The Folin–Ciocalteu assay, which is a standardized method used routinely to measure the polyphenolic content in foods, beverages, and dietary supplements was used to investigate GB. The extract was found to have 59.91 ± 2.17 mg/L GAE (Fig. 1). The scavenging activity of GB was assessed by a colorimetric assay of a methanol solution of cell-free DPPH free radical assay, which is a standard approach to evaluate antioxidants^19^. Here, GB was found to have a scavenging IC_50_ value of 110.75 µg/ml (Fig. 2), which is considered to be potent. Next, GB was examined for its capacity to protect cells undergoing H_2_O_2_-induced oxidative stress and death. Cells were pretreated with two concentrations of GB for 18 h. GB has been shown to express plasma membrane impermeable molecules like SOD and catalase, which have the capacity to degrade H_2_O_2_^6^. Therefore, in order to remove their extracellular residual presence, a wash step with fresh warm (37 °C) media was included after the GB treatment. Subsequently, cells were exposed for an additional 24 h to a final concentration of 600 µM of H_2_O_2_ and their viability was examined *via* propidium iodide (PI) exclusion and flow cytometry. The criterion utilized to designate a dead cell was its loss of plasma membrane integrity, as previously reported^45^. Results indicate that low amount of GB extract (10 µl) showed moderate cytoprotection upon H_2_O_2_-induced oxidative stress. However, 50 µl of GB showed significant protection activity (*P* < 0.01) on YT, pre-B Nalm-6 and Jurkat cells (Fig. 3). These findings suggest that GB possess significant antioxidative protection in cells undergoing strong aggression inflicted by H_2_O_2_ in a concentration-dependent modality. Moreover, GB itself exhibited no cytotoxic activity at the concentrations used after 24 h of cell exposure. The results presented here are in agreement with the idea that plant products could be used as an option in antioxidative preventive medicine.

The activation of various signaling pathways was investigated to explain the mechanism used by GB in mitigating oxidative stress and reducing cell death. Initially, pre-B Nalm-6 cells were treated with GB and the phosphorylation of a panel of the Src family of tyrosine kinases (SFKs) was investigated. SFKs plays crucial roles in ample intracellular signal transduction. They mediate redundant as well as unique functions involving cell metabolism, activation, differentiation, proliferation, migration, and apoptosis among others^46^. The main SFKs member expressed in B cell lineages is Lyn, which has both positive and negative regulatory functions upon stimulation of a variety of signaling pathways^47^. In the intracellular space as a membrane-associated tyrosine kinase, Lyn is crucial for the regulation of B cell activation while the cytosolic Lyn cleaved soluble fragment can act as an inhibitor of apoptosis^46–48^. Lyn has been reported to be over-expressed in chronic lymphocytic leukemia (CLL) cells where it blocks apoptosis^49^. Here, our findings revealed that GB treatment of pre-B Nalm-6 cells results in the phosphorylation of Lyn (Tyr397) in a concentration-depended modality that is potentially implicated in the antioxidative mechanism that protects the lymphoid cells (Fig. 4). In line with this data, it has been demonstrated that Lyn and Fgr kinases possess anti-apoptotic activities in the human promyelocytic leukemia cell line (HL-60)^50^. Additionally, Lyn mediated tumor progression and cell motility in a head and neck squamous cell carcinoma model *in vivo*^51^. Moreover, in glioblastoma, Lyn contributed with a prominent increased activity facilitating cell survival^52^. These studies suggest that Lyn could be functioning as a protective anti-apoptotic kinase, making cancer cells more resistant to the anti-cancer immune response and/or to anti-cancer drugs.

Once activated, Lyn can phosphorylate signaling molecules leading to the activation of various signaling pathways, including PI3K^53^. In every tissue, numerous biological processes are regulated by the PI3K pathway. An important effector of PI3K signaling is Akt, also known as protein kinase B (PKB). Akt is a serine/threonine kinase that plays a crucial role in transducing PI3K cell survival signals^54^. Overexpression of a constitutively active Akt in lymphocytes in *in vivo* transgenic mice results in improvement of lymphocyte survival by preventing sepsis-induced apoptosis^55^. Consistent with these observations, thymocytes from fetal thymus organ primary culture of animals overexpressing Akt showed an increment in viability upon γ-radiation, dexamethasone, and Fas ligand exposure^56^. In addition, activation of Akt in Nalm-6 cells, has been shown to decrease the percentage of apoptotic cells induced by two clinically used anticancer drugs, vincristine and adriamycin^57^. Moreover, activation of the Lyn/PI3K/Akt pathway was found to be involved in the survival and proliferation of myeloma cells and resistance to apoptosis in juvenile myelomonocytic leukemia (JMML)^58,59^. In line with these studies, our findings revealed that GB treatment results in phosphorylation of Akt (both Thr308 and Ser473) in a pre-B Nalm-6 cell line in a time-dependent fashion (Fig. 5). The activation of this protein by GB treatment suggests that the Lyn/PI3K/Akt signaling pathway might be implicated in its cytoprotection mechanism.

Another well-recognized signaling pathway that can be activated by Lyn is MAPK/ERK, also involved in cell proliferation, differentiation, and survival^49,60^. MAPK/ERK activation leads to upregulation of cell cycle genes and blocking of apoptosis in CLL^49^. Also, both the MAPK/ERK and the PI3K/Akt pathways have been found to be essential for the survival of several breast cancer cell lines^61^. In this study, it was found that GB is able to induce phosphorylation/activation of ERK in a time-regulated mode (Fig. 5). Thus, in addition to the GB-mediated activation of Lyn/PI3K/Akt pathway, GB is also able to trigger the MAPK/ERK pathway and both contribute as cell pro-survival mechanisms, which potentially lead at least in part, to the GB cytoprotective effect. In addition to these pathways, several other biochemical pathways may participate in the mitigation of cytotoxicity in pre-B cells undergoing aggressive oxidative stress. In the future, it will be necessary to identify the survival-associated downstream targets affected by GB-mediated Lyn, MAPK/ERK, and Akt phosphorylation/activation.

Because of its role in promoting proliferation and survival of lymphoid cells, activation of the transcription factor STAT5 in response to GB was also assessed. STAT5 promotes the transcription of anti-apoptotic and cell cycle progression genes and was recently found to have a protective role against oxidative stress^31,62^. Inhibition of STAT5 expression in lymphoid pre-B cell lines, Nalm-6, 697 and Reh, lead to increased FAS-elicited cell death, and higher spontaneous apoptosis^31^. Here, treatment of Nalm-6 cells with GB resulted in a slight increase in STAT5 phosphorylation, indicating that GB confers its protective signals independently of the STAT5 cascade (Fig. 5). Therefore, under the circumstances tested, it appears that the contribution of the anti-apoptotic JAK2-STAT5 signaling pathway is unimportant as a protective mechanism elicited by GB; however, this pathway could have a potential additive or synergistic effect in preventing cell death concurrently with Lyn/PI3K/Akt and MAPK/ERK pathways. In addition to these pathways, several other biochemical pathways may participate in the mitigation of cytotoxicity in pre-B cells undergoing aggressive oxidative stress. In the future, it will be necessary to identify the survival-associated downstream targets affected by GB-mediated Lyn, MAPK/ERK, and Akt phosphorylation/activation.

Hence, based on the evidence presented here, GB contains phenolic compounds that exhibit potent free radical scavenging activities. Additionally, GB exerted a cytoprotective effect on human lymphoid cells, as evidenced by a significant reduction in cell death after oxidative stress exposure. Findings at the molecular level suggest that GB activated the pro-survival Lyn/PI3K/Akt and MAPK/ERK signaling pathways and these occurrences could be implicated in conveying its cytoprotective effect in human lymphoid cells. Our study describes a unique set of functional signaling transduction pathways and mechanisms of action of GB in lymphoid pre-B Nalm-6 cells, involved in mitigating cell death after oxidative stress. Therefore, it should be judicious to evaluate the GB-mediated properties in an animal model, as an herbal option comprising its antioxidant and cytoprotective activity.
